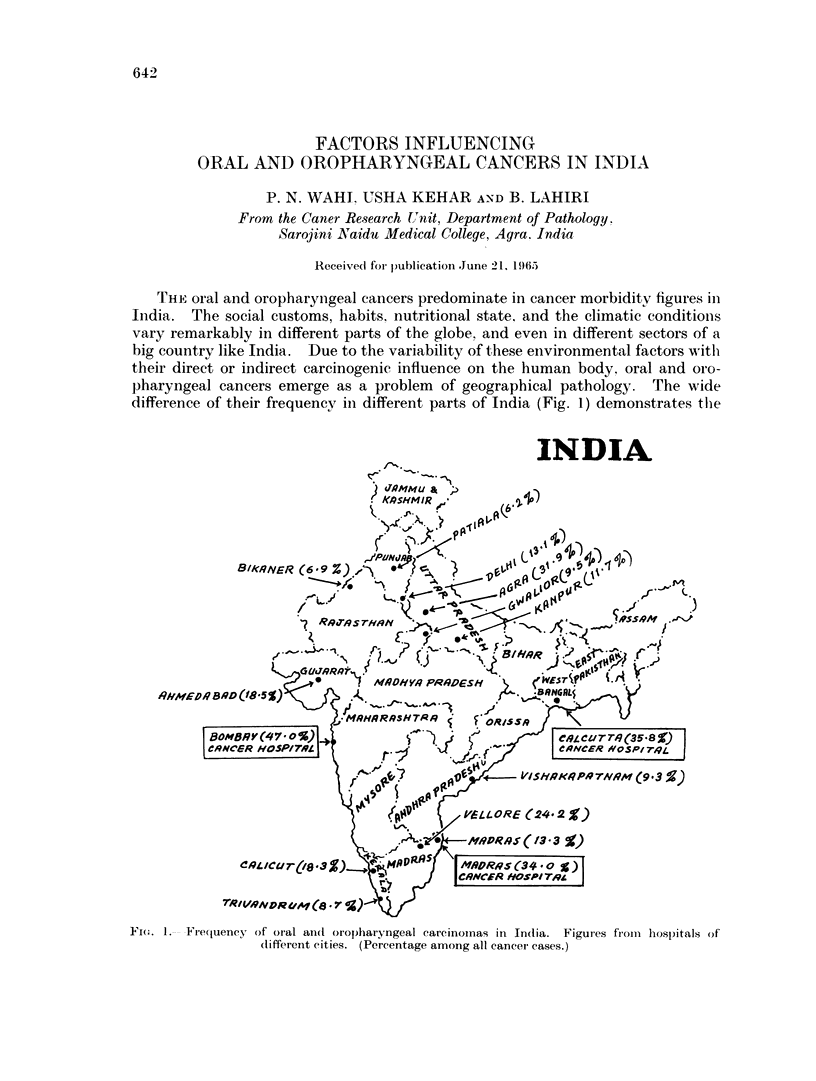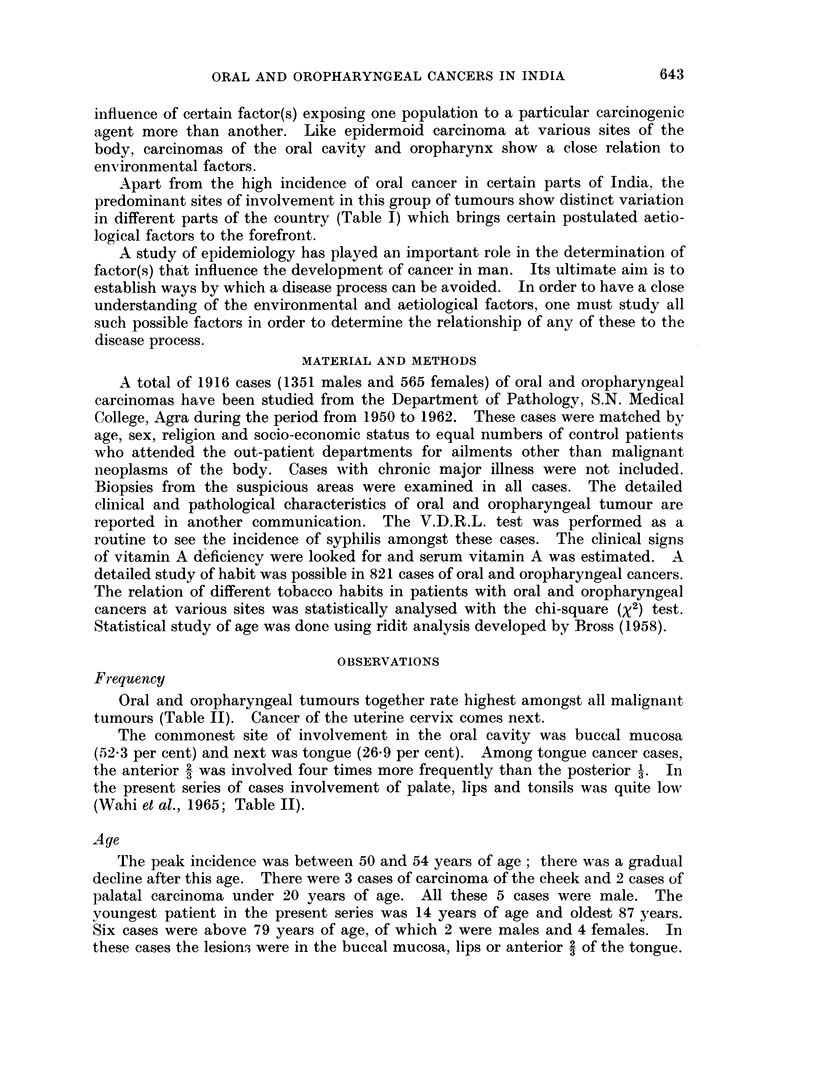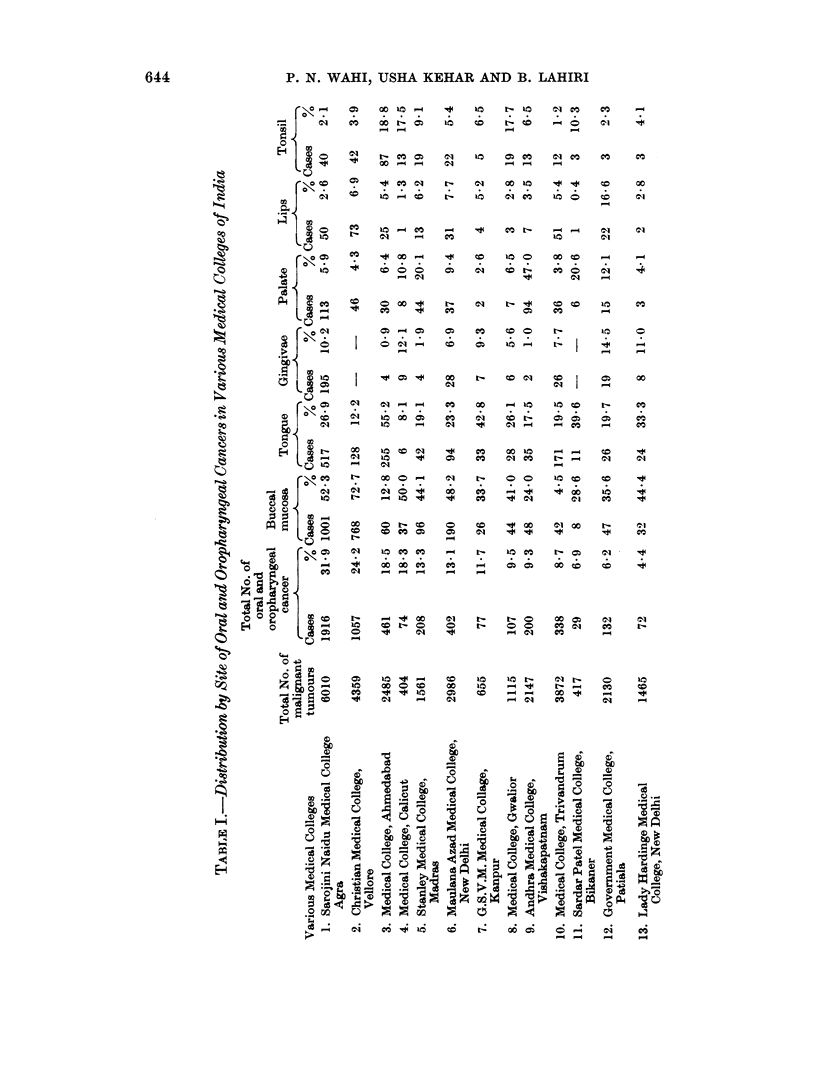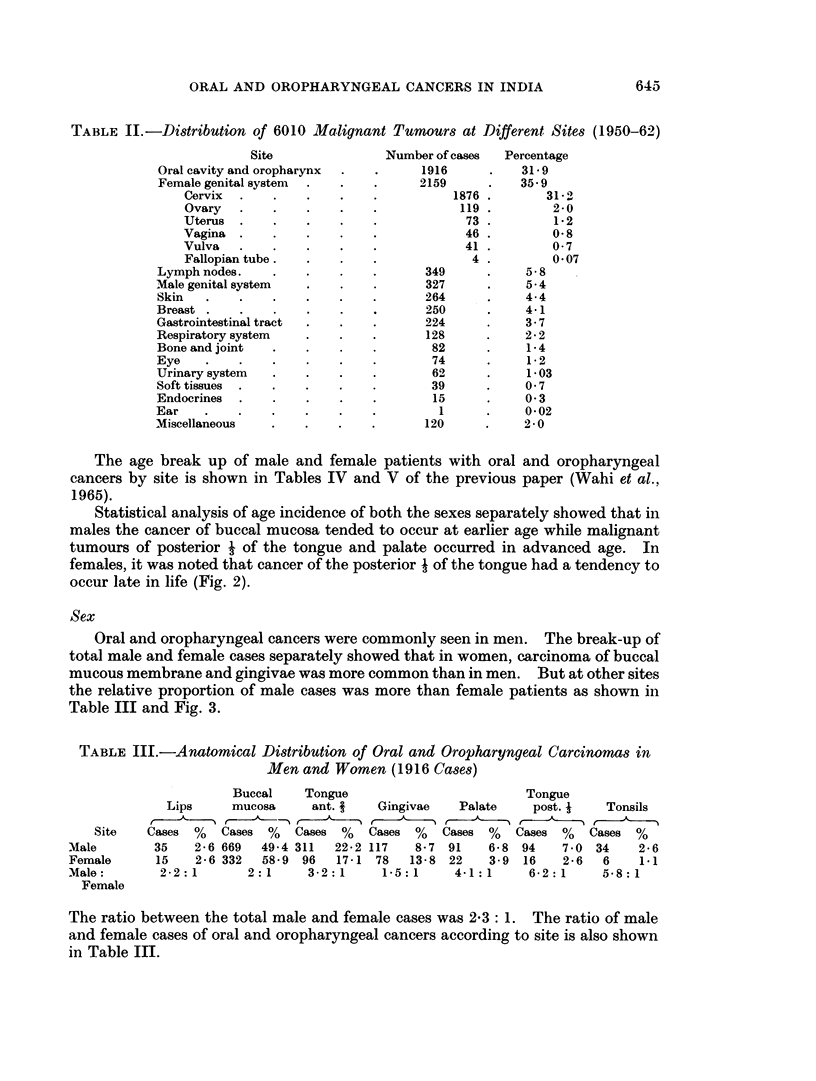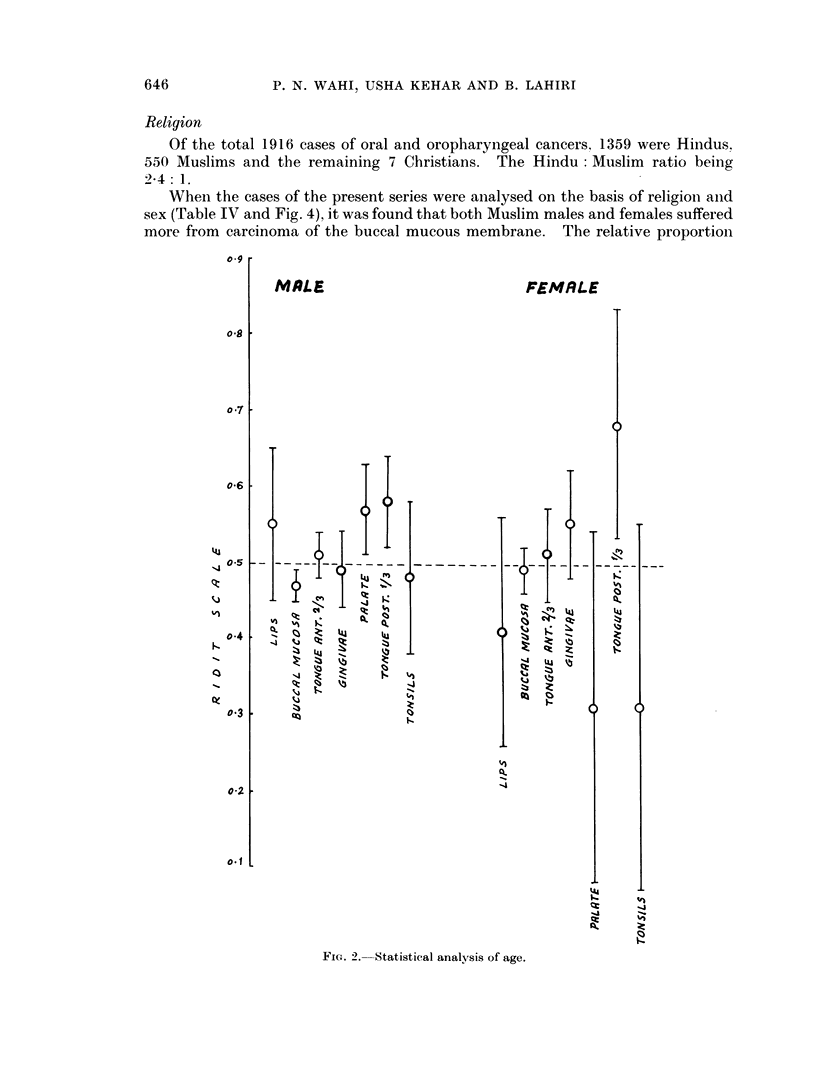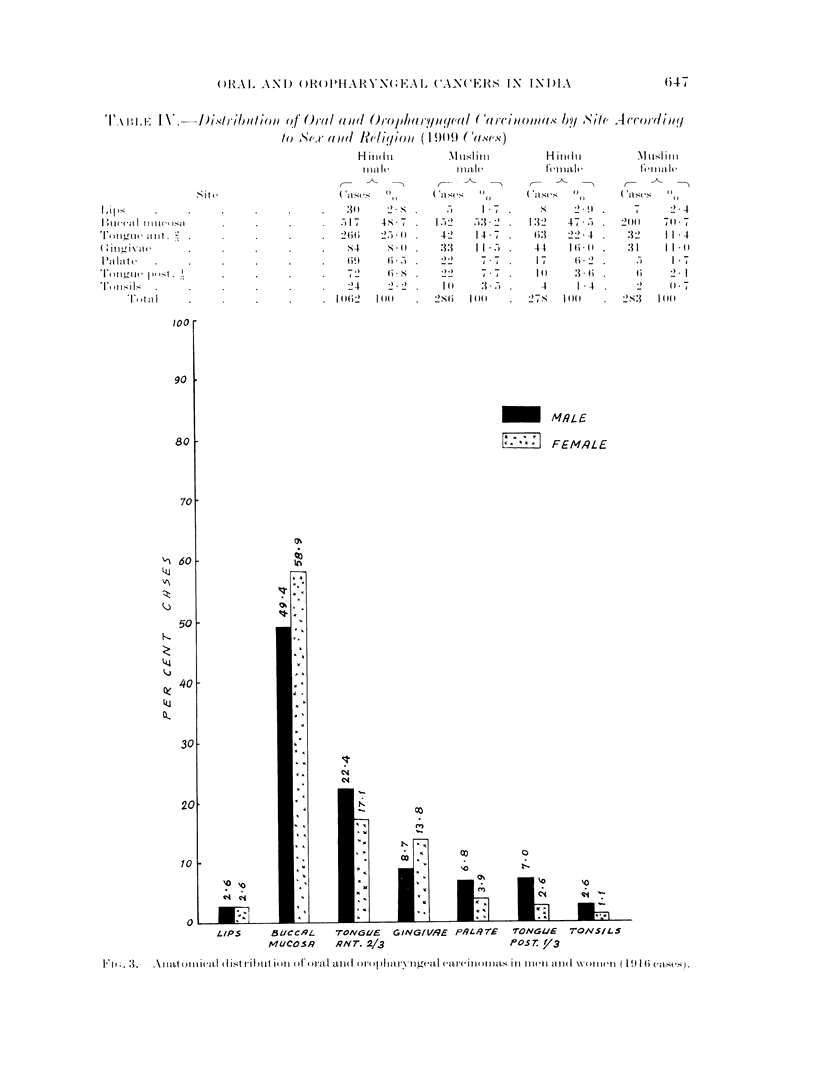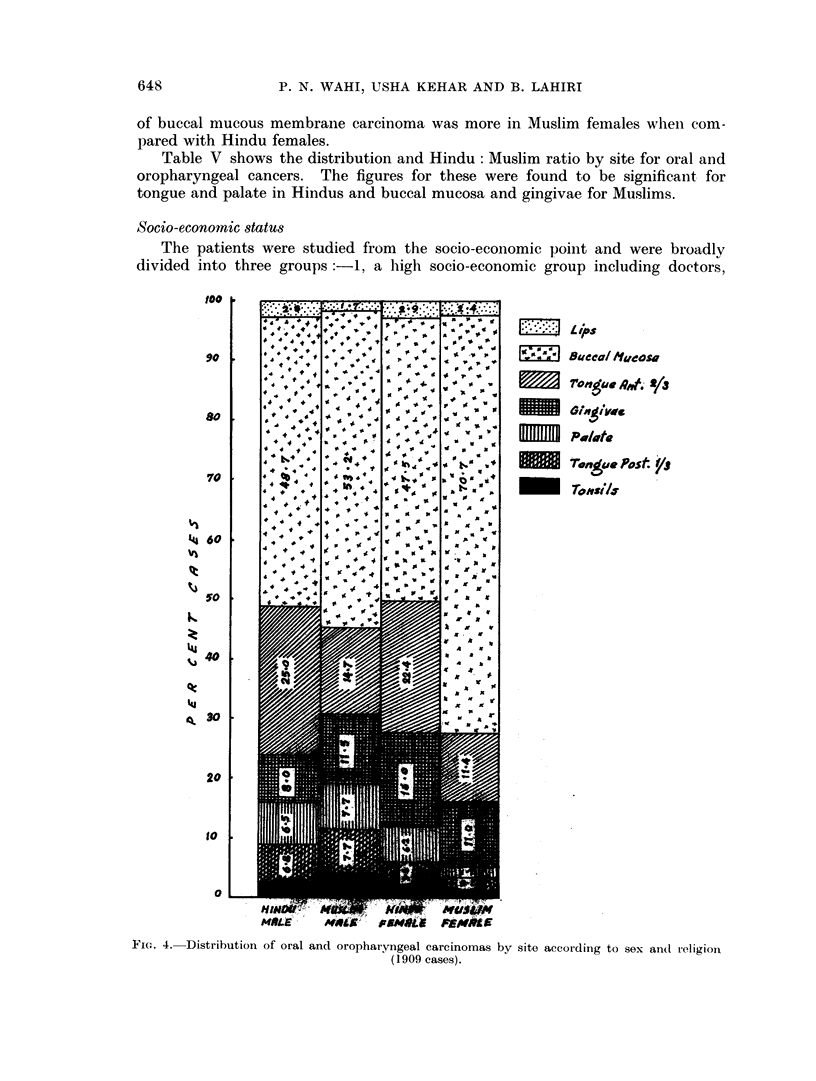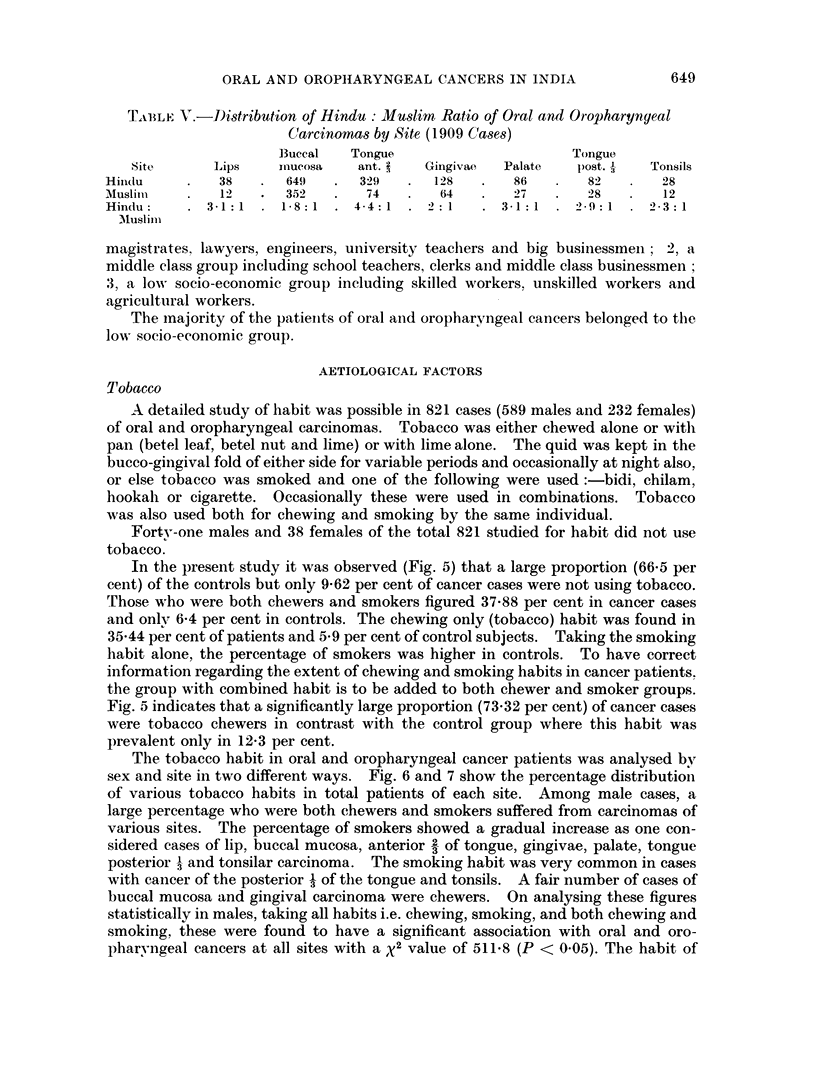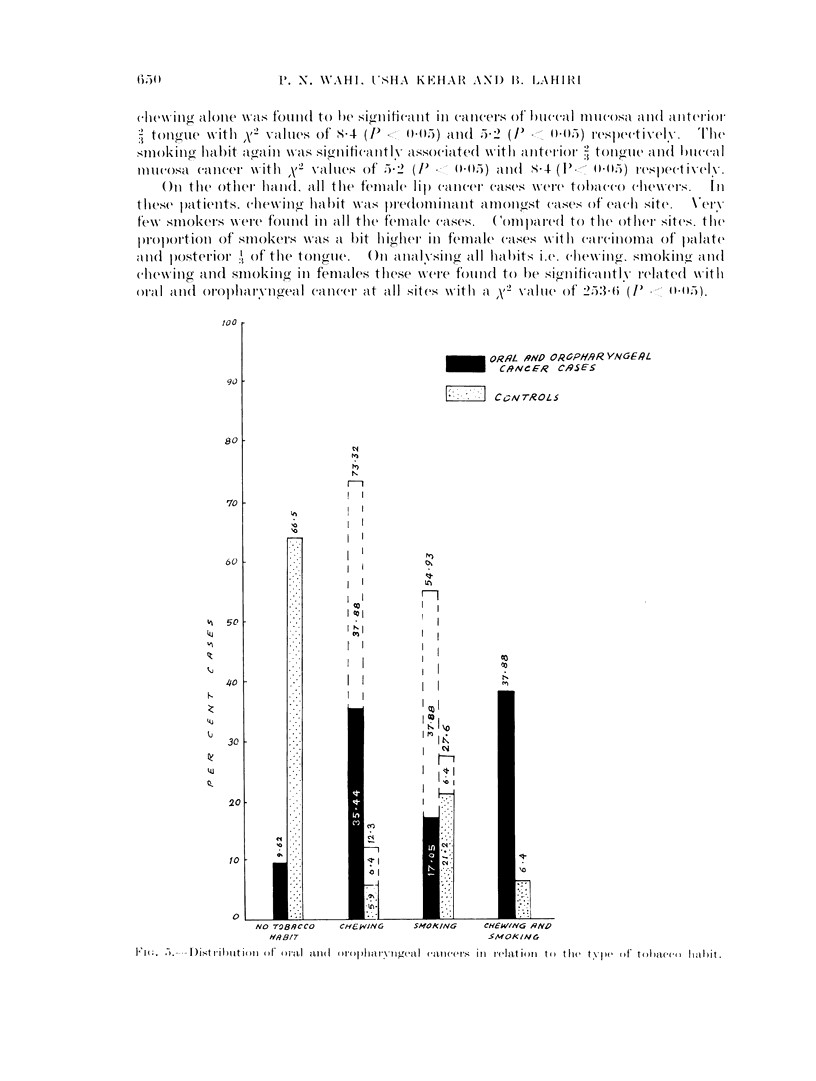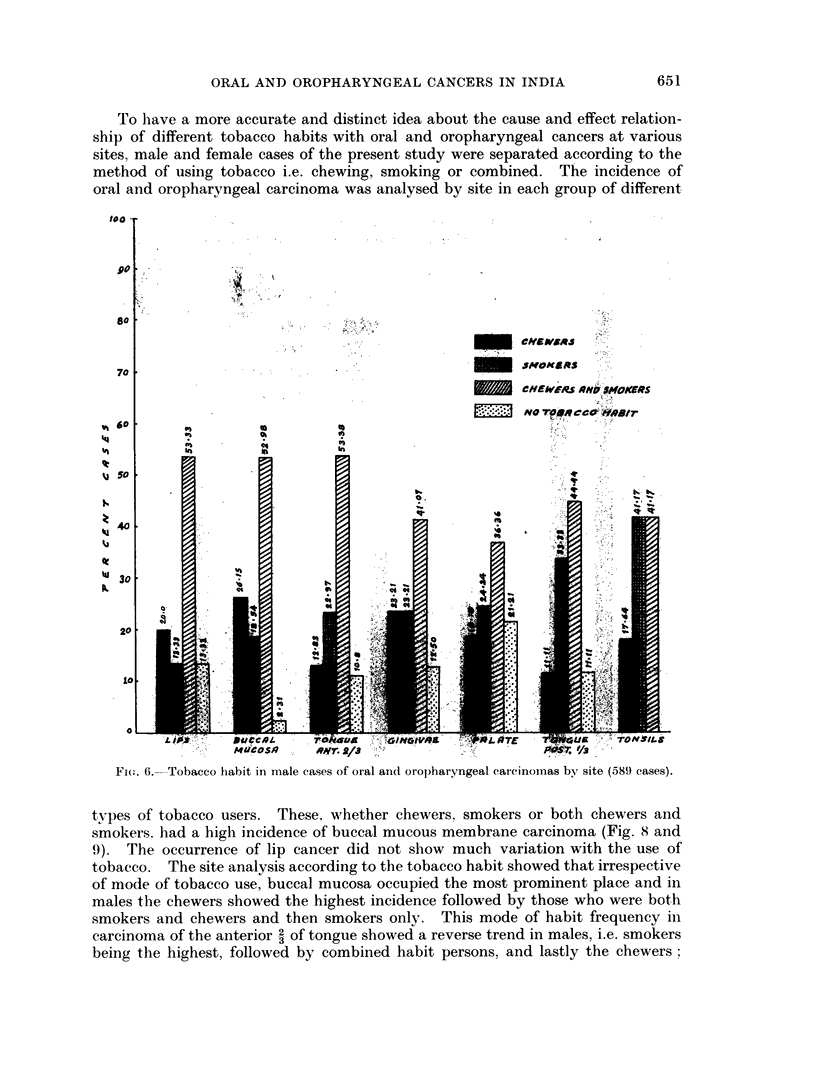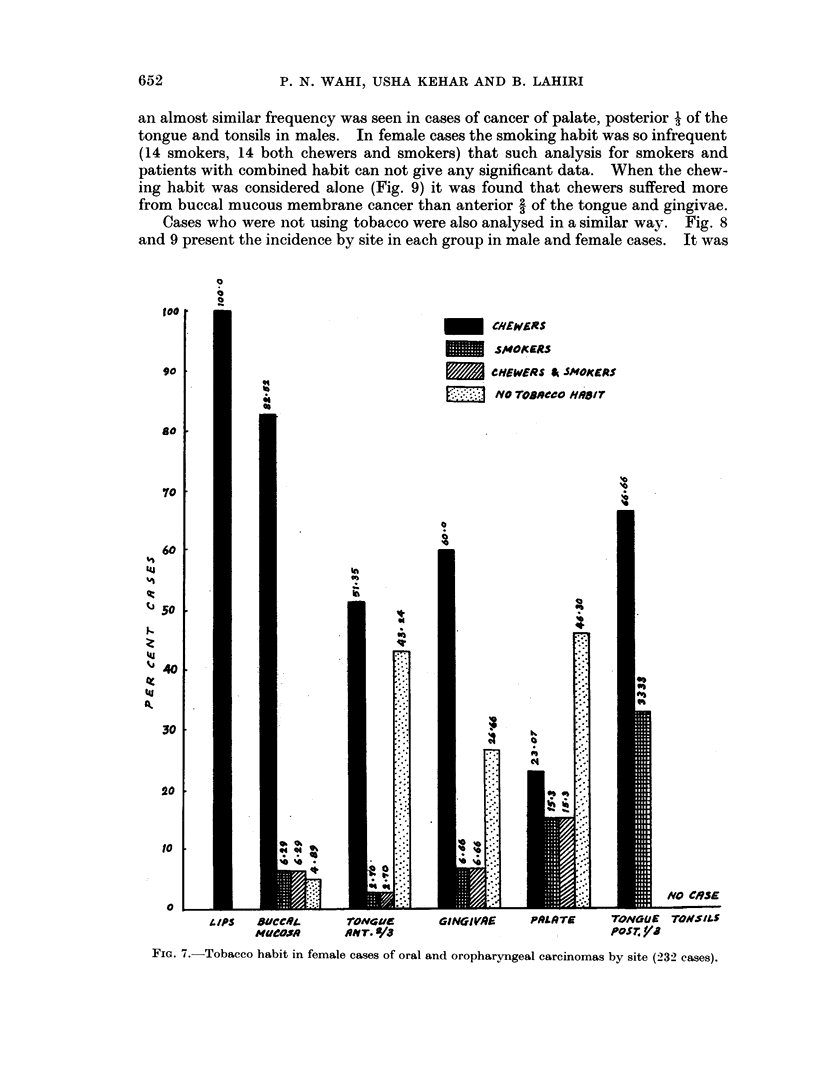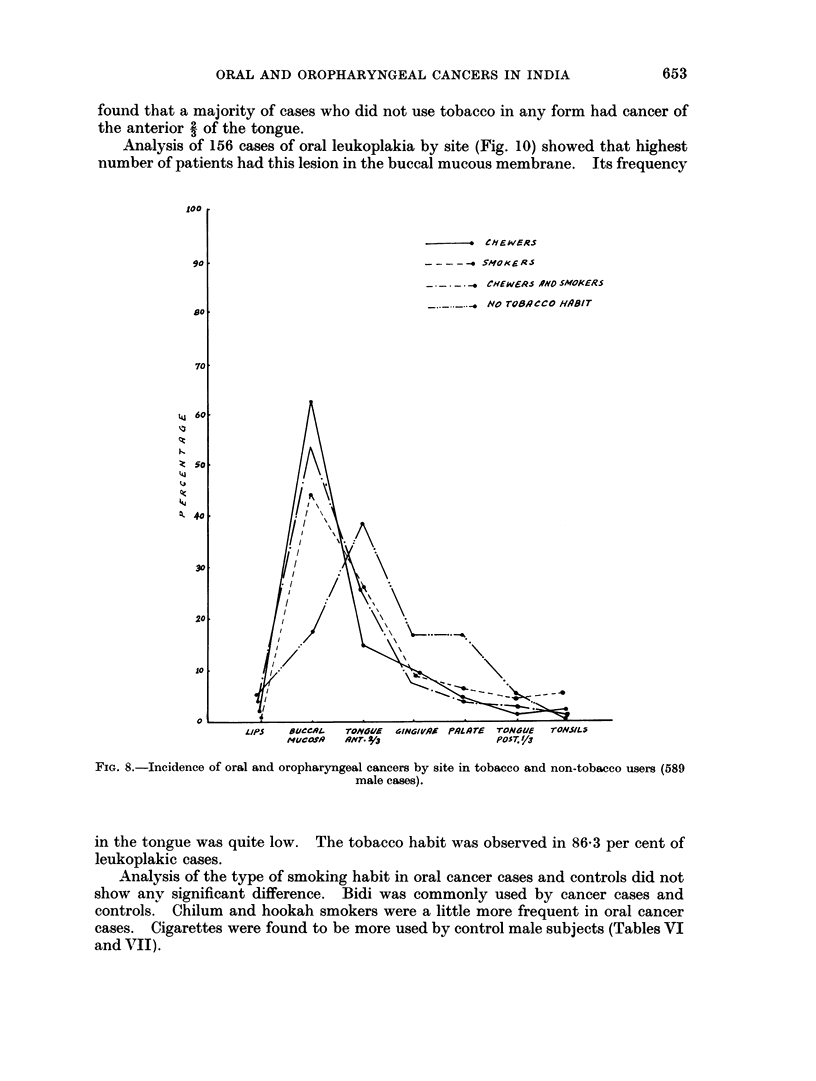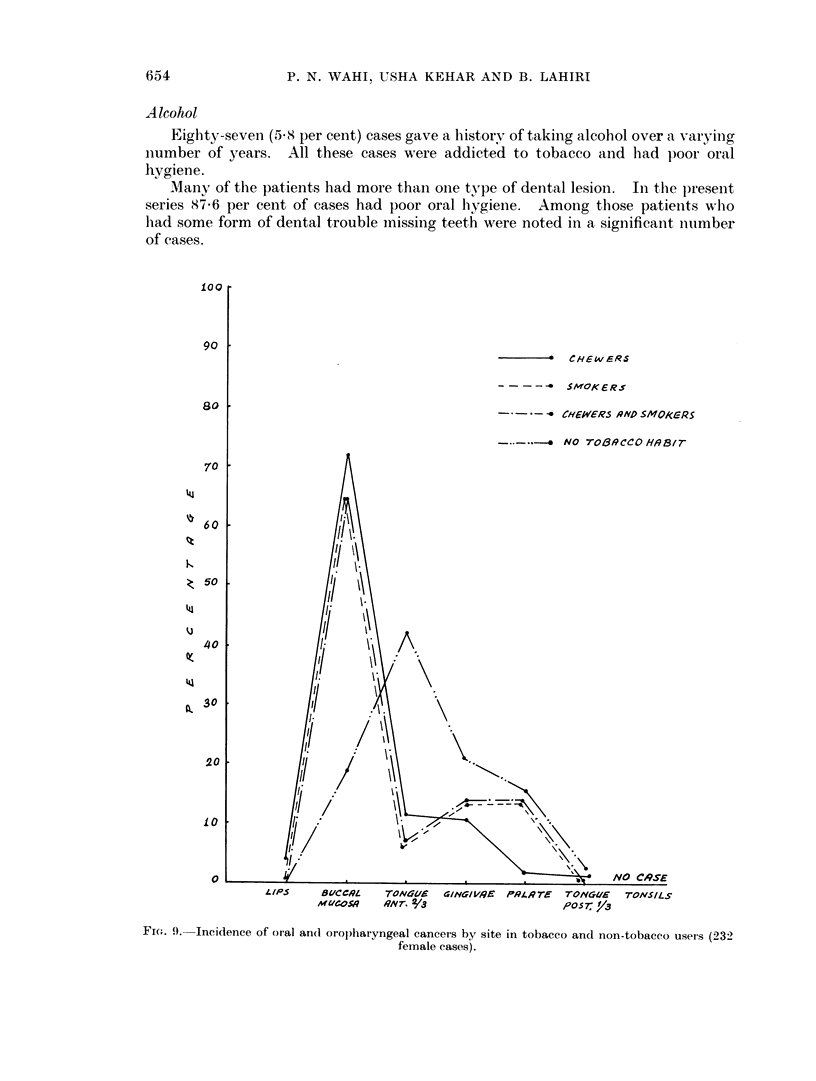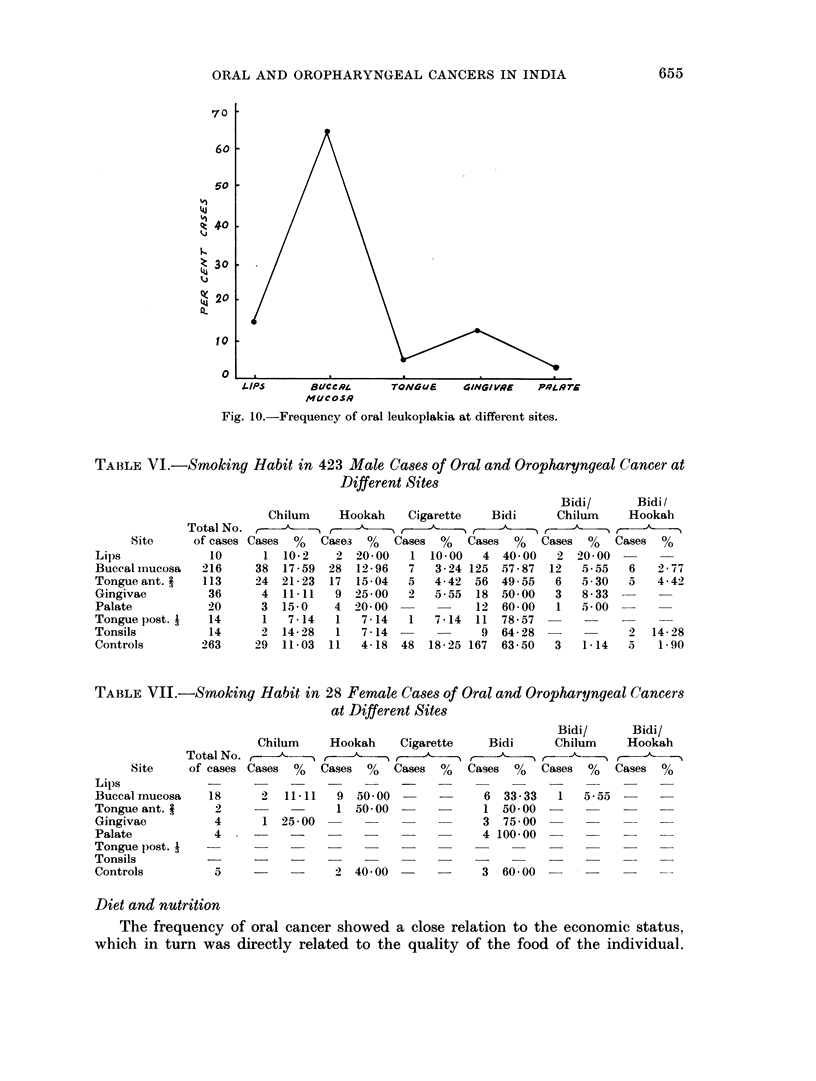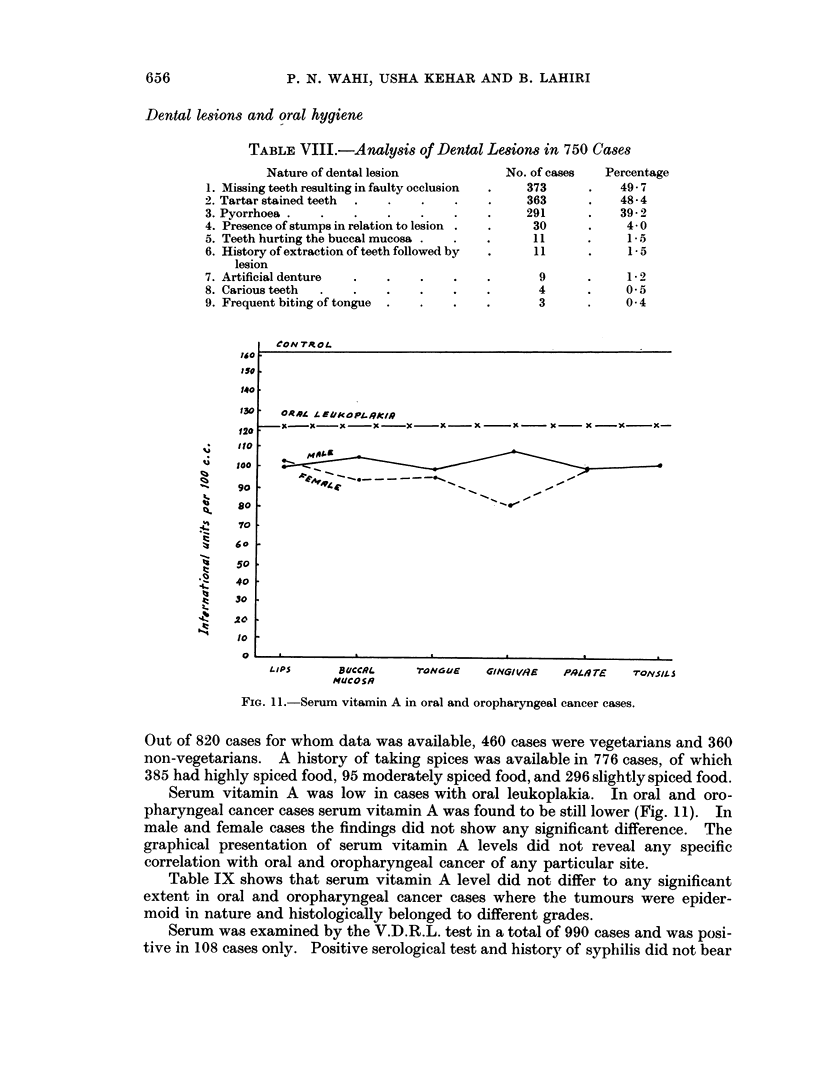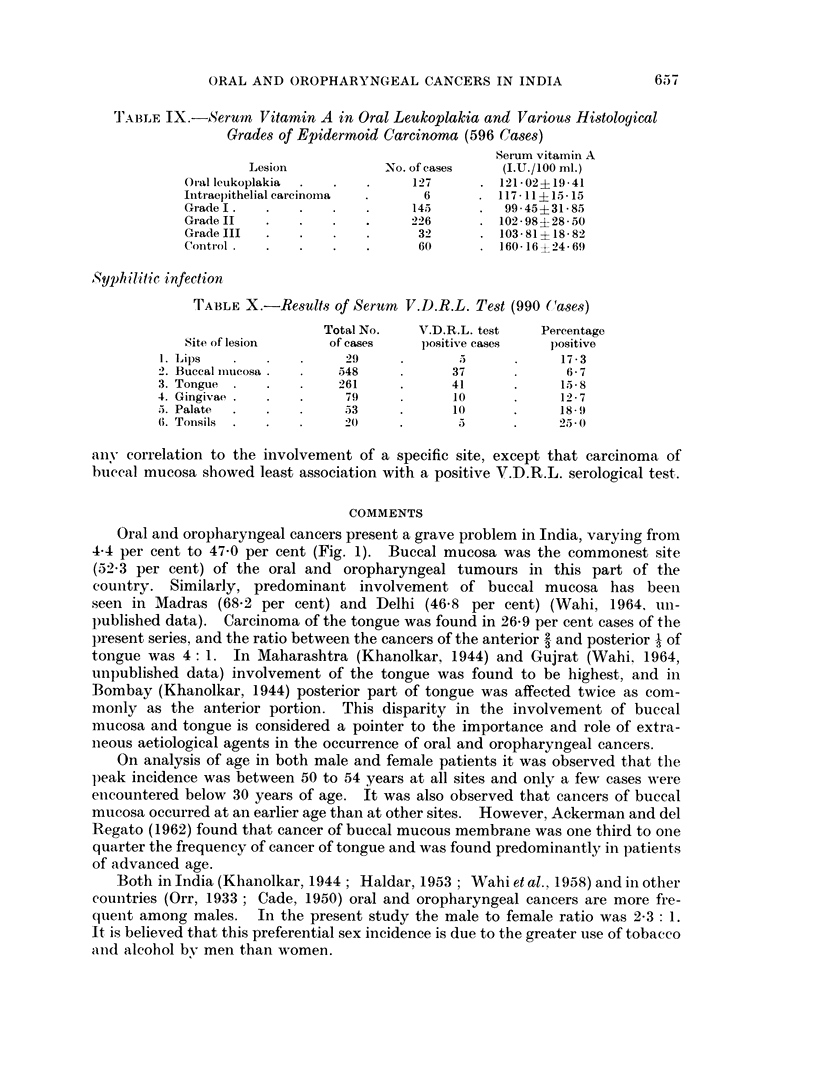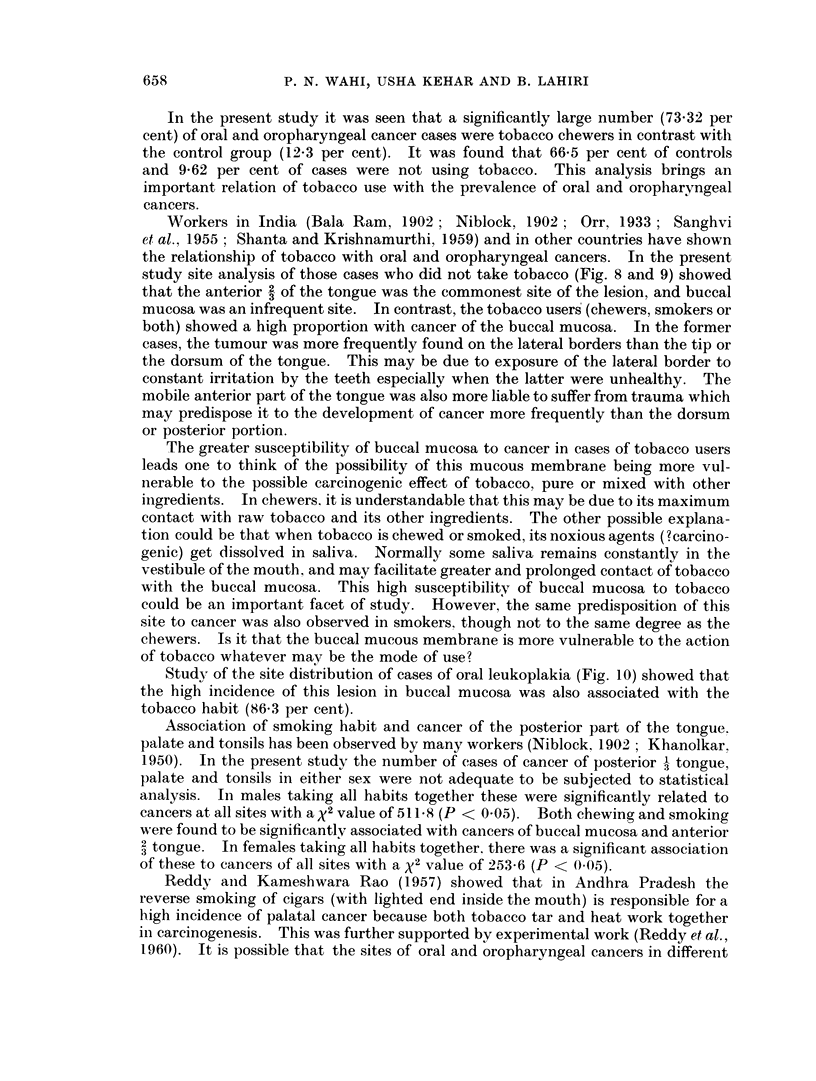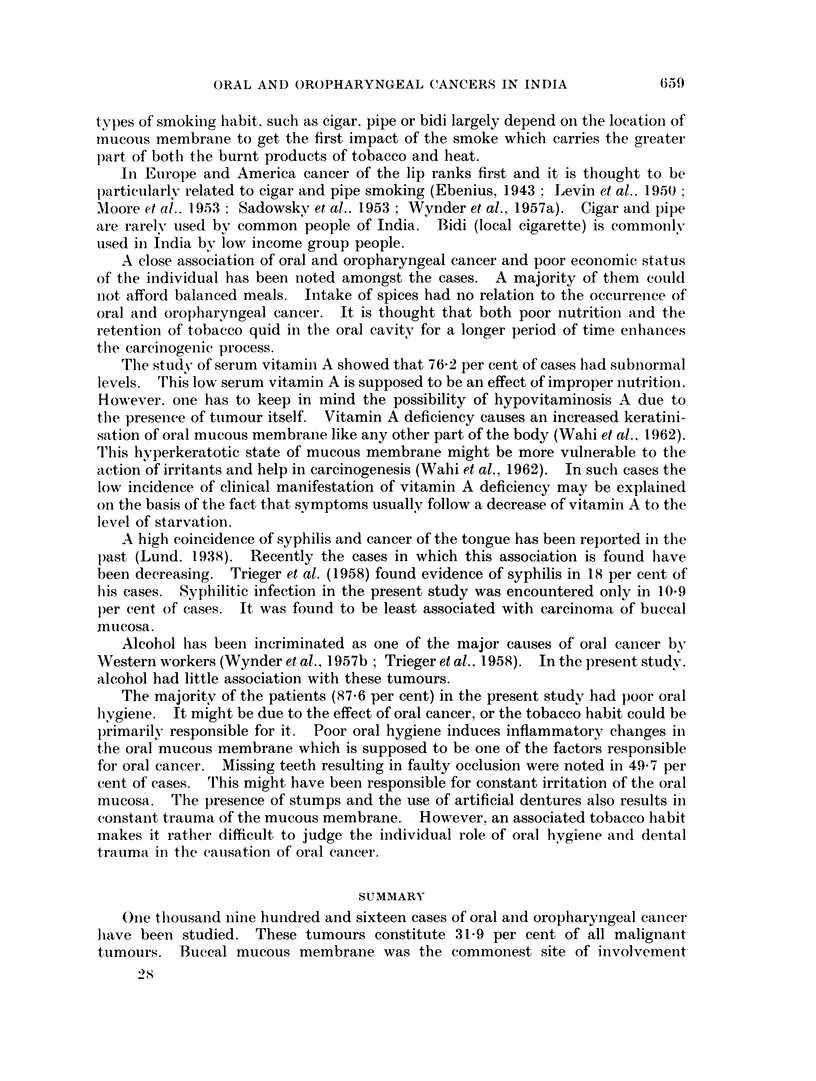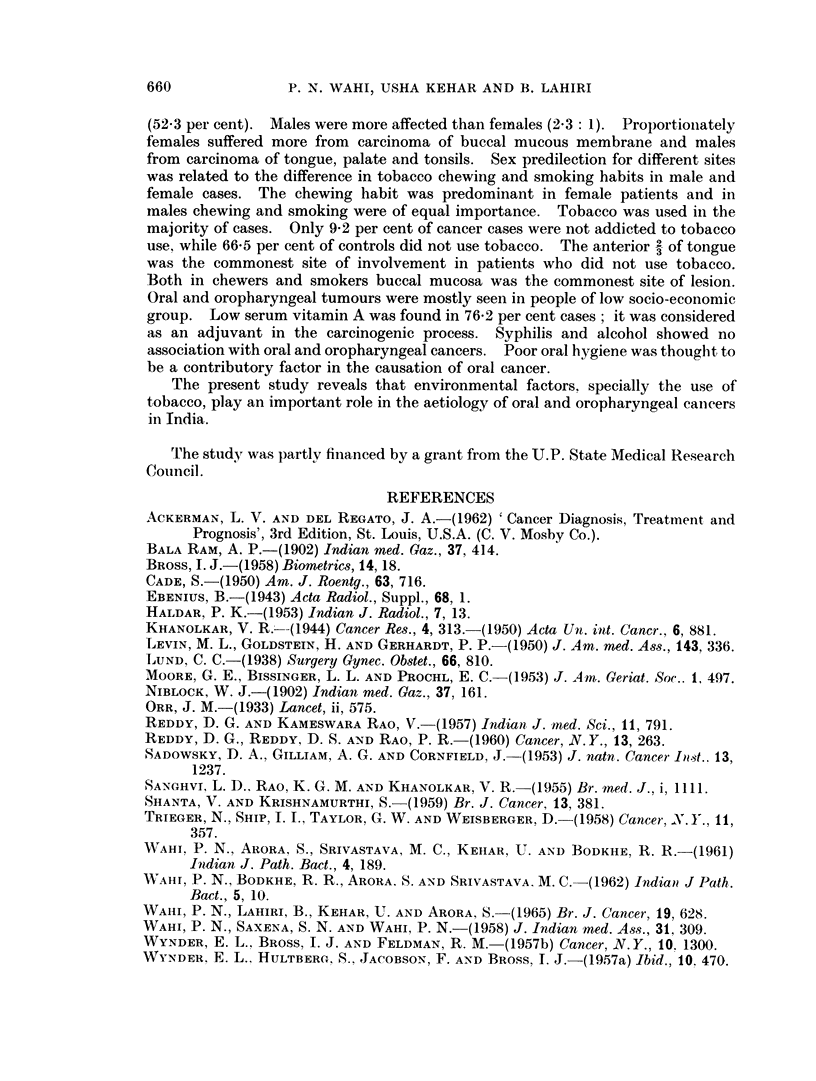# Factors influencing oral and oropharyngeal cancers in India.

**DOI:** 10.1038/bjc.1965.80

**Published:** 1965-12

**Authors:** P. N. Wahi, U. Kehar, B. Lahiri


### FACTORS INFLUENCING ORAL AND OROPHARYNGEAL CANCERS IN INDIA

P. N. WAHI, USHA KEHAR AND B. LAHIRI

*From the Caner Research Unit, Department of Pathology, Sarojini Naidu Medical College, Agra, India*

Received for publication June 21, 1965

THE oral and oropharyngeal cancers predominate in cancer morbidity figures in India. The social customs, habits, nutritional state, and the climatic conditions vary remarkably in different parts of the globe, and even in different sectors of a big country like India. Due to the variability of these environmental factors with their direct or indirect carcinogenic influence on the human body, oral and oropharyngeal cancers emerge as a problem of geographical pathology. The wide difference of their frequency in different parts of India (Fig. 1) demonstrates the

FIG. 1.—Frequency of oral and oropharyngeal carcinomas in India. Figures from hospitals of different cities. (Percentage among all cancer cases.)

influence of certain factor(s) exposing one population to a particular carcinogenic agent more than another. Like epidermoid carcinoma at various sites of the body, carcinomas of the oral cavity and oropharynx show a close relation to environmental factors.

Apart from the high incidence of oral cancer in certain parts of India, the predominant sites of involvement in this group of tumours show distinct variation in different parts of the country (Table I) which brings certain postulated aetiological factors to the forefront.

A study of epidemiology has played an important role in the determination of factor(s) that influence the development of cancer in man. Its ultimate aim is to establish ways by which a disease process can be avoided. In order to have a close understanding of the environmental and aetiological factors, one must study all such possible factors in order to determine the relationship of any of these to the disease process.

### MATERIAL AND METHODS

A total of 1916 cases (1351 males and 565 females) of oral and oropharyngeal carcinomas have been studied from the Department of Pathology, S.N. Medical College, Agra during the period from 1950 to 1962. These cases were matched by age, sex, religion and socio-economic status to equal numbers of control patients who attended the out-patient departments for ailments other than malignant neoplasms of the body. Cases with chronic major illness were not included. Biopsies from the suspicious areas were examined in all cases. The detailed clinical and pathological characteristics of oral and oropharyngeal tumour are reported in another communication. The V.D.R.L. test was performed as a routine to see the incidence of syphilis amongst these cases. The clinical signs of vitamin A deficiency were looked for and serum vitamin A was estimated. A detailed study of habit was possible in 821 cases of oral and oropharyngeal cancers. The relation of different tobacco habits in patients with oral and oropharyngeal cancers at various sites was statistically analysed with the chi-square ($\chi^2$) test. Statistical study of age was done using ridit analysis developed by Bross (1958).

### OBSERVATIONS

### *Frequency*

Oral and oropharyngeal tumours together rate highest amongst all malignant tumours (Table II). Cancer of the uterine cervix comes next.

The commonest site of involvement in the oral cavity was buccal mucosa (52·3 per cent) and next was tongue (26·9 per cent). Among tongue cancer cases, the anterior $\frac{2}{3}$ was involved four times more frequently than the posterior $\frac{1}{3}$. In the present series of cases involvement of palate, lips and tonsils was quite low (Wahi *et al.*, 1965; Table II).

### *Age*

The peak incidence was between 50 and 54 years of age ; there was a gradual decline after this age. There were 3 cases of carcinoma of the cheek and 2 cases of palatal carcinoma under 20 years of age. All these 5 cases were male. The youngest patient in the present series was 14 years of age and oldest 87 years. Six cases were above 79 years of age, of which 2 were males and 4 females. In these cases the lesions were in the buccal mucosa, lips or anterior $\frac{2}{3}$ of the tongue.

TABLE I.—*Distribution by Site of Oral and Oropharyngeal Cancers in Various Medical Colleges of India*

| Various Medical Colleges | Total No. of malignant tumours | Total No. of oral and oropharyngeal cancer | | Buccal mucosa | | Tongue | | Gingivae | | Palate | | Lips | | Tonsil | |
|---|---|---|---|---|---|---|---|---|---|---|---|---|---|---|---|
| | | Cases | % | Cases | % | Cases | % | Cases | % | Cases | % | Cases | % | Cases | % |
| 1. Sarojini Naidu Medical College Agra | 6010 | 1916 | 31·9 | 1001 | 52·3 | 517 | 26·9 | 195 | 10·2 | 113 | 5·9 | 50 | 2·6 | 40 | 2·1 |
| 2. Christian Medical College, Vellore | 4359 | 1057 | 24·2 | 768 | 72·7 | 128 | 12·2 | — | — | 46 | 4·3 | 73 | 6·9 | 42 | 3·9 |
| 3. Medical College, Ahmedabad | 2485 | 461 | 18·5 | 60 | 12·8 | 255 | 55·2 | 4 | 0·9 | 30 | 6·4 | 25 | 5·4 | 87 | 18·8 |
| 4. Medical College, Calicut | 404 | 74 | 18·3 | 37 | 50·0 | 6 | 8·1 | 9 | 12·1 | 8 | 10·8 | 1 | 1·3 | 13 | 17·5 |
| 5. Stanley Medical College, Madras | 1561 | 208 | 13·3 | 96 | 44·1 | 42 | 19·1 | 4 | 1·9 | 44 | 20·1 | 13 | 6·2 | 19 | 9·1 |
| 6. Maulana Azad Medical College, New Delhi | 2986 | 402 | 13·1 | 190 | 48·2 | 94 | 23·3 | 28 | 6·9 | 37 | 9·4 | 31 | 7·7 | 22 | 5·4 |
| 7. G.S.V.M. Medical Collage, Kanpur | 655 | 77 | 11·7 | 26 | 33·7 | 33 | 42·8 | 7 | 9·3 | 2 | 2·6 | 4 | 5·2 | 5 | 6·5 |
| 8. Medical College, Gwalior | 1115 | 107 | 9·5 | 44 | 41·0 | 28 | 26·1 | 6 | 5·6 | 7 | 6·5 | 3 | 2·8 | 19 | 17·7 |
| 9. Andhra Medical College, Vishakapatnam | 2147 | 200 | 9·3 | 48 | 24·0 | 35 | 17·5 | 2 | 1·0 | 94 | 47·0 | 7 | 3·5 | 13 | 6·5 |
| 10. Medical College, Trivandrum | 3872 | 338 | 8·7 | 42 | 4·5 | 171 | 19·5 | 26 | 7·7 | 36 | 3·8 | 51 | 5·4 | 12 | 1·2 |
| 11. Sardar Patel Medical College, Bikaner | 417 | 29 | 6·9 | 8 | 28·6 | 11 | 39·6 | — | — | 6 | 20·6 | 1 | 0·4 | 3 | 10·3 |
| 12. Government Medical College, Patiala | 2130 | 132 | 6·2 | 47 | 35·6 | 26 | 19·7 | 19 | 14·5 | 15 | 12·1 | 22 | 16·6 | 3 | 2·3 |
| 13. Lady Hardinge Medical College, New Delhi | 1465 | 72 | 4·4 | 32 | 44·4 | 24 | 33·3 | 8 | 11·0 | 3 | 4·1 | 2 | 2·8 | 3 | 4·1 |

TABLE II.—*Distribution of* 6010 *Malignant Tumours at Different Sites* (1950–62)

| Site | Number of cases | Percentage |
|---|---|---|
| Oral cavity and oropharynx | 1916 | 31·9 |
| Female genital system | 2159 | 35·9 |
| Cervix | 1876 | 31·2 |
| Ovary | 119 | 2·0 |
| Uterus | 73 | 1·2 |
| Vagina | 46 | 0·8 |
| Vulva | 41 | 0·7 |
| Fallopian tube | 4 | 0·07 |
| Lymph nodes | 349 | 5·8 |
| Male genital system | 327 | 5·4 |
| Skin | 264 | 4·4 |
| Breast | 250 | 4·1 |
| Gastrointestinal tract | 224 | 3·7 |
| Respiratory system | 128 | 2·2 |
| Bone and joint | 82 | 1·4 |
| Eye | 74 | 1·2 |
| Urinary system | 62 | 1·03 |
| Soft tissues | 39 | 0·7 |
| Endocrines | 15 | 0·3 |
| Ear | 1 | 0·02 |
| Miscellaneous | 120 | 2·0 |

The age break up of male and female patients with oral and oropharyngeal cancers by site is shown in Tables IV and V of the previous paper (Wahi *et al.*, 1965).

Statistical analysis of age incidence of both the sexes separately showed that in males the cancer of buccal mucosa tended to occur at earlier age while malignant tumours of posterior $\frac{1}{3}$ of the tongue and palate occurred in advanced age. In females, it was noted that cancer of the posterior $\frac{1}{3}$ of the tongue had a tendency to occur late in life (Fig. 2).

*Sex*

Oral and oropharyngeal cancers were commonly seen in men. The break-up of total male and female cases separately showed that in women, carcinoma of buccal mucous membrane and gingivae was more common than in men. But at other sites the relative proportion of male cases was more than female patients as shown in Table III and Fig. 3.

TABLE III.—*Anatomical Distribution of Oral and Oropharyngeal Carcinomas in Men and Women* (1916 *Cases*)

| | Lips | | Buccal mucosa | | Tongue ant. $\frac{2}{3}$ | | Gingivae | | Palate | | Tongue post. $\frac{1}{3}$ | | Tonsils | |
|---|---|---|---|---|---|---|---|---|---|---|---|---|---|---|
| Site | Cases | % | Cases | % | Cases | % | Cases | % | Cases | % | Cases | % | Cases | % |
| Male | 35 | 2·6 | 669 | 49·4 | 311 | 22·2 | 117 | 8·7 | 91 | 6·8 | 94 | 7·0 | 34 | 2·6 |
| Female | 15 | 2·6 | 332 | 58·9 | 96 | 17·1 | 78 | 13·8 | 22 | 3·9 | 16 | 2·6 | 6 | 1·1 |
| Male : Female | 2·2 : 1 | | 2 : 1 | | 3·2 : 1 | | 1·5 : 1 | | 4·1 : 1 | | 6·2 : 1 | | 5·8 : 1 | |

The ratio between the total male and female cases was 2·3 : 1. The ratio of male and female cases of oral and oropharyngeal cancers according to site is also shown in Table III.

*Religion*

Of the total 1916 cases of oral and oropharyngeal cancers, 1359 were Hindus, 550 Muslims and the remaining 7 Christians. The Hindu : Muslim ratio being 2·4 : 1.

When the cases of the present series were analysed on the basis of religion and sex (Table IV and Fig. 4), it was found that both Muslim males and females suffered more from carcinoma of the buccal mucous membrane. The relative proportion

FIG. 2.—Statistical analysis of age.

TABLE IV.—*Distribution of Oral and Oropharyngeal Carcinomas by Site According to Sex and Religion* (1909 *Cases*)

| Site | Hindu male | | Muslim male | | Hindu female | | Muslim female | |
|---|---|---|---|---|---|---|---|---|
| | Cases | % | Cases | % | Cases | % | Cases | % |
| Lips | 30 | 2·8 | 5 | 1·7 | 8 | 2·9 | 7 | 2·4 |
| Buccal mucosa | 517 | 48·7 | 152 | 53·2 | 132 | 47·5 | 200 | 70·7 |
| Tongue ant. $\frac{2}{3}$ | 266 | 25·0 | 42 | 14·7 | 63 | 22·4 | 32 | 11·4 |
| Gingivae | 84 | 8·0 | 33 | 11·5 | 44 | 16·0 | 31 | 11·0 |
| Palate | 69 | 6·5 | 22 | 7·7 | 17 | 6·2 | 5 | 1·7 |
| Tongue post. $\frac{1}{3}$ | 72 | 6·8 | 22 | 7·7 | 10 | 3·6 | 6 | 2·1 |
| Tonsils | 24 | 2·2 | 10 | 3·5 | 4 | 1·4 | 2 | 0·7 |
| Total | 1062 | 100 | 286 | 100 | 278 | 100 | 283 | 100 |

FIG. 3.—Anatomical distribution of oral and oropharyngeal carcinomas in men and women (1916 cases).

of buccal mucous membrane carcinoma was more in Muslim females when compared with Hindu females.

Table V shows the distribution and Hindu : Muslim ratio by site for oral and oropharyngeal cancers. The figures for these were found to be significant for tongue and palate in Hindus and buccal mucosa and gingivae for Muslims.

*Socio-economic status*

The patients were studied from the socio-economic point and were broadly divided into three groups :—1, a high socio-economic group including doctors,

FIG. 4.—Distribution of oral and oropharyngeal carcinomas by site according to sex and religion (1909 cases).

TABLE V.—*Distribution of Hindu : Muslim Ratio of Oral and Oropharyngeal Carcinomas by Site* (1909 *Cases*)

| Site | Lips | Buccal mucosa | Tongue ant. $\frac{2}{3}$ | Gingivae | Palate | Tongue post. $\frac{1}{3}$ | Tonsils |
|---|---|---|---|---|---|---|---|
| Hindu | 38 | 649 | 329 | 128 | 86 | 82 | 28 |
| Muslim | 12 | 352 | 74 | 64 | 27 | 28 | 12 |
| Hindu : Muslim | 3·1 : 1 | 1·8 : 1 | 4·4 : 1 | 2 : 1 | 3·1 : 1 | 2·9 : 1 | 2·3 : 1 |

magistrates, lawyers, engineers, university teachers and big businessmen ; 2, a middle class group including school teachers, clerks and middle class businessmen ; 3, a low socio-economic group including skilled workers, unskilled workers and agricultural workers.

The majority of the patients of oral and oropharyngeal cancers belonged to the low socio-economic group.

### AETIOLOGICAL FACTORS

### *Tobacco*

A detailed study of habit was possible in 821 cases (589 males and 232 females) of oral and oropharyngeal carcinomas. Tobacco was either chewed alone or with pan (betel leaf, betel nut and lime) or with lime alone. The quid was kept in the bucco-gingival fold of either side for variable periods and occasionally at night also, or else tobacco was smoked and one of the following were used :—bidi, chilam, hookah or cigarette. Occasionally these were used in combinations. Tobacco was also used both for chewing and smoking by the same individual.

Forty-one males and 38 females of the total 821 studied for habit did not use tobacco.

In the present study it was observed (Fig. 5) that a large proportion (66·5 per cent) of the controls but only 9·62 per cent of cancer cases were not using tobacco. Those who were both chewers and smokers figured 37·88 per cent in cancer cases and only 6·4 per cent in controls. The chewing only (tobacco) habit was found in 35·44 per cent of patients and 5·9 per cent of control subjects. Taking the smoking habit alone, the percentage of smokers was higher in controls. To have correct information regarding the extent of chewing and smoking habits in cancer patients, the group with combined habit is to be added to both chewer and smoker groups. Fig. 5 indicates that a significantly large proportion (73·32 per cent) of cancer cases were tobacco chewers in contrast with the control group where this habit was prevalent only in 12·3 per cent.

The tobacco habit in oral and oropharyngeal cancer patients was analysed by sex and site in two different ways. Fig. 6 and 7 show the percentage distribution of various tobacco habits in total patients of each site. Among male cases, a large percentage who were both chewers and smokers suffered from carcinomas of various sites. The percentage of smokers showed a gradual increase as one considered cases of lip, buccal mucosa, anterior $\frac{2}{3}$ of tongue, gingivae, palate, tongue posterior $\frac{1}{3}$ and tonsilar carcinoma. The smoking habit was very common in cases with cancer of the posterior $\frac{1}{3}$ of the tongue and tonsils. A fair number of cases of buccal mucosa and gingival carcinoma were chewers. On analysing these figures statistically in males, taking all habits i.e. chewing, smoking, and both chewing and smoking, these were found to have a significant association with oral and oropharyngeal cancers at all sites with a $\chi^2$ value of 511·8 ($P < 0{\cdot}05$). The habit of chewing alone was found to be significant in cancers of buccal mucosa and anterior $\frac{2}{3}$ tongue with $\chi^2$ values of 8·4 ($P < 0·05$) and 5·2 ($P < 0·05$) respectively. The smoking habit again was significantly associated with anterior $\frac{2}{3}$ tongue and buccal mucosa cancer with $\chi^2$ values of 5·2 ($P < 0·05$) and 8·4 ($P < 0·05$) respectively.

On the other hand, all the female lip cancer cases were tobacco chewers. In these patients, chewing habit was predominant amongst cases of each site. Very few smokers were found in all the female cases. Compared to the other sites, the proportion of smokers was a bit higher in female cases with carcinoma of palate and posterior $\frac{1}{3}$ of the tongue. On analysing all habits i.e. chewing, smoking and chewing and smoking in females these were found to be significantly related with oral and oropharyngeal cancer at all sites with a $\chi^2$ value of 253·6 ($P < 0·05$).

FIG. 5.—Distribution of oral and oropharyngeal cancers in relation to the type of tobacco habit.

To have a more accurate and distinct idea about the cause and effect relationship of different tobacco habits with oral and oropharyngeal cancers at various sites, male and female cases of the present study were separated according to the method of using tobacco i.e. chewing, smoking or combined. The incidence of oral and oropharyngeal carcinoma was analysed by site in each group of different

FIG. 6.—Tobacco habit in male cases of oral and oropharyngeal carcinomas by site (589 cases).

types of tobacco users. These, whether chewers, smokers or both chewers and smokers, had a high incidence of buccal mucous membrane carcinoma (Fig. 8 and 9). The occurrence of lip cancer did not show much variation with the use of tobacco. The site analysis according to the tobacco habit showed that irrespective of mode of tobacco use, buccal mucosa occupied the most prominent place and in males the chewers showed the highest incidence followed by those who were both smokers and chewers and then smokers only. This mode of habit frequency in carcinoma of the anterior $\frac{2}{3}$ of tongue showed a reverse trend in males, i.e. smokers being the highest, followed by combined habit persons, and lastly the chewers ; an almost similar frequency was seen in cases of cancer of palate, posterior ⅓ of the tongue and tonsils in males. In female cases the smoking habit was so infrequent (14 smokers, 14 both chewers and smokers) that such analysis for smokers and patients with combined habit can not give any significant data. When the chewing habit was considered alone (Fig. 9) it was found that chewers suffered more from buccal mucous membrane cancer than anterior ⅔ of the tongue and gingivae.

Cases who were not using tobacco were also analysed in a similar way. Fig. 8 and 9 present the incidence by site in each group in male and female cases. It was

Fig. 7.—Tobacco habit in female cases of oral and oropharyngeal carcinomas by site (232 cases).

found that a majority of cases who did not use tobacco in any form had cancer of the anterior ⅔ of the tongue.

Analysis of 156 cases of oral leukoplakia by site (Fig. 10) showed that highest number of patients had this lesion in the buccal mucous membrane. Its frequency

FIG. 8.—Incidence of oral and oropharyngeal cancers by site in tobacco and non-tobacco users (589 male cases).

in the tongue was quite low. The tobacco habit was observed in 86·3 per cent of leukoplakic cases.

Analysis of the type of smoking habit in oral cancer cases and controls did not show any significant difference. Bidi was commonly used by cancer cases and controls. Chilum and hookah smokers were a little more frequent in oral cancer cases. Cigarettes were found to be more used by control male subjects (Tables VI and VII).

*Alcohol*

Eighty-seven (5·8 per cent) cases gave a history of taking alcohol over a varying number of years. All these cases were addicted to tobacco and had poor oral hygiene.

Many of the patients had more than one type of dental lesion. In the present series 87·6 per cent of cases had poor oral hygiene. Among those patients who had some form of dental trouble missing teeth were noted in a significant number of cases.

FIG. 9.—Incidence of oral and oropharyngeal cancers by site in tobacco and non-tobacco users (232 female cases).

Fig. 10.—Frequency of oral leukoplakia at different sites.

TABLE VI.—*Smoking Habit in* 423 *Male Cases of Oral and Oropharyngeal Cancer at Different Sites*

| Site | Total No. of cases | Chilum Cases | Chilum % | Hookah Cases | Hookah % | Cigarette Cases | Cigarette % | Bidi Cases | Bidi % | Bidi/Chilum Cases | Bidi/Chilum % | Bidi/Hookah Cases | Bidi/Hookah % |
|---|---|---|---|---|---|---|---|---|---|---|---|---|---|
| Lips | 10 | 1 | 10·2 | 2 | 20·00 | 1 | 10·00 | 4 | 40·00 | 2 | 20·00 | — | — |
| Buccal mucosa | 216 | 38 | 17·59 | 28 | 12·96 | 7 | 3·24 | 125 | 57·87 | 12 | 5·55 | 6 | 2·77 |
| Tongue ant. ⅔ | 113 | 24 | 21·23 | 17 | 15·04 | 5 | 4·42 | 56 | 49·55 | 6 | 5·30 | 5 | 4·42 |
| Gingivae | 36 | 4 | 11·11 | 9 | 25·00 | 2 | 5·55 | 18 | 50·00 | 3 | 8·33 | — | — |
| Palate | 20 | 3 | 15·0 | 4 | 20·00 | — | — | 12 | 60·00 | 1 | 5·00 | — | — |
| Tongue post. ⅓ | 14 | 1 | 7·14 | 1 | 7·14 | 1 | 7·14 | 11 | 78·57 | — | — | — | — |
| Tonsils | 14 | 2 | 14·28 | 1 | 7·14 | — | — | 9 | 64·28 | — | — | 2 | 14·28 |
| Controls | 263 | 29 | 11·03 | 11 | 4·18 | 48 | 18·25 | 167 | 63·50 | 3 | 1·14 | 5 | 1·90 |

TABLE VII.—*Smoking Habit in* 28 *Female Cases of Oral and Oropharyngeal Cancers at Different Sites*

| Site | Total No. of cases | Chilum Cases | Chilum % | Hookah Cases | Hookah % | Cigarette Cases | Cigarette % | Bidi Cases | Bidi % | Bidi/Chilum Cases | Bidi/Chilum % | Bidi/Hookah Cases | Bidi/Hookah % |
|---|---|---|---|---|---|---|---|---|---|---|---|---|---|
| Lips | — | — | — | — | — | — | — | — | — | — | — | — | — |
| Buccal mucosa | 18 | 2 | 11·11 | 9 | 50·00 | — | — | 6 | 33·33 | 1 | 5·55 | — | — |
| Tongue ant. ⅔ | 2 | — | — | 1 | 50·00 | — | — | 1 | 50·00 | — | — | — | — |
| Gingivae | 4 | 1 | 25·00 | — | — | — | — | 3 | 75·00 | — | — | — | — |
| Palate | 4 | — | — | — | — | — | — | 4 | 100·00 | — | — | — | — |
| Tongue post. ⅓ | — | — | — | — | — | — | — | — | — | — | — | — | — |
| Tonsils | — | — | — | — | — | — | — | — | — | — | — | — | — |
| Controls | 5 | — | — | 2 | 40·00 | — | — | 3 | 60·00 | — | — | — | — |

*Diet and nutrition*

The frequency of oral cancer showed a close relation to the economic status, which in turn was directly related to the quality of the food of the individual.

*Dental lesions and oral hygiene*

TABLE VIII.—*Analysis of Dental Lesions in* 750 *Cases*

| Nature of dental lesion | No. of cases | Percentage |
|---|---|---|
| 1. Missing teeth resulting in faulty occlusion | 373 | 49·7 |
| 2. Tartar stained teeth | 363 | 48·4 |
| 3. Pyorrhoea | 291 | 39·2 |
| 4. Presence of stumps in relation to lesion | 30 | 4·0 |
| 5. Teeth hurting the buccal mucosa | 11 | 1·5 |
| 6. History of extraction of teeth followed by lesion | 11 | 1·5 |
| 7. Artificial denture | 9 | 1·2 |
| 8. Carious teeth | 4 | 0·5 |
| 9. Frequent biting of tongue | 3 | 0·4 |

FIG. 11.—Serum vitamin A in oral and oropharyngeal cancer cases.

Out of 820 cases for whom data was available, 460 cases were vegetarians and 360 non-vegetarians. A history of taking spices was available in 776 cases, of which 385 had highly spiced food, 95 moderately spiced food, and 296 slightly spiced food.

Serum vitamin A was low in cases with oral leukoplakia. In oral and oropharyngeal cancer cases serum vitamin A was found to be still lower (Fig. 11). In male and female cases the findings did not show any significant difference. The graphical presentation of serum vitamin A levels did not reveal any specific correlation with oral and oropharyngeal cancer of any particular site.

Table IX shows that serum vitamin A level did not differ to any significant extent in oral and oropharyngeal cancer cases where the tumours were epidermoid in nature and histologically belonged to different grades.

Serum was examined by the V.D.R.L. test in a total of 990 cases and was positive in 108 cases only. Positive serological test and history of syphilis did not bear

TABLE IX.—*Serum Vitamin A in Oral Leukoplakia and Various Histological Grades of Epidermoid Carcinoma* (596 *Cases*)

| Lesion | No. of cases | Serum vitamin A (I.U./100 ml.) |
|---|---|---|
| Oral leukoplakia | 127 | 121·02±19·41 |
| Intraepithelial carcinoma | 6 | 117·11±15·15 |
| Grade I | 145 | 99·45±31·85 |
| Grade II | 226 | 102·98±28·50 |
| Grade III | 32 | 103·81±18·82 |
| Control | 60 | 160·16±24·69 |

*Syphilitic infection*

TABLE X.—*Results of Serum V.D.R.L. Test* (990 *Cases*)

| Site of lesion | Total No. of cases | V.D.R.L. test positive cases | Percentage positive |
|---|---|---|---|
| 1. Lips | 29 | 5 | 17·3 |
| 2. Buccal mucosa | 548 | 37 | 6·7 |
| 3. Tongue | 261 | 41 | 15·8 |
| 4. Gingivae | 79 | 10 | 12·7 |
| 5. Palate | 53 | 10 | 18·9 |
| 6. Tonsils | 20 | 5 | 25·0 |

any correlation to the involvement of a specific site, except that carcinoma of buccal mucosa showed least association with a positive V.D.R.L. serological test.

### COMMENTS

Oral and oropharyngeal cancers present a grave problem in India, varying from 4·4 per cent to 47·0 per cent (Fig. 1). Buccal mucosa was the commonest site (52·3 per cent) of the oral and oropharyngeal tumours in this part of the country. Similarly, predominant involvement of buccal mucosa has been seen in Madras (68·2 per cent) and Delhi (46·8 per cent) (Wahi, 1964, unpublished data). Carcinoma of the tongue was found in 26·9 per cent cases of the present series, and the ratio between the cancers of the anterior $\frac{2}{3}$ and posterior $\frac{1}{3}$ of tongue was 4 : 1. In Maharashtra (Khanolkar, 1944) and Gujrat (Wahi, 1964, unpublished data) involvement of the tongue was found to be highest, and in Bombay (Khanolkar, 1944) posterior part of tongue was affected twice as commonly as the anterior portion. This disparity in the involvement of buccal mucosa and tongue is considered a pointer to the importance and role of extraneous aetiological agents in the occurrence of oral and oropharyngeal cancers.

On analysis of age in both male and female patients it was observed that the peak incidence was between 50 to 54 years at all sites and only a few cases were encountered below 30 years of age. It was also observed that cancers of buccal mucosa occurred at an earlier age than at other sites. However, Ackerman and del Regato (1962) found that cancer of buccal mucous membrane was one third to one quarter the frequency of cancer of tongue and was found predominantly in patients of advanced age.

Both in India (Khanolkar, 1944 ; Haldar, 1953 ; Wahi *et al.*, 1958) and in other countries (Orr, 1933 ; Cade, 1950) oral and oropharyngeal cancers are more frequent among males. In the present study the male to female ratio was 2·3 : 1. It is believed that this preferential sex incidence is due to the greater use of tobacco and alcohol by men than women.

In the present study it was seen that a significantly large number (73·32 per cent) of oral and oropharyngeal cancer cases were tobacco chewers in contrast with the control group (12·3 per cent). It was found that 66·5 per cent of controls and 9·62 per cent of cases were not using tobacco. This analysis brings an important relation of tobacco use with the prevalence of oral and oropharyngeal cancers.

Workers in India (Bala Ram, 1902; Niblock, 1902; Orr, 1933; Sanghvi *et al.*, 1955; Shanta and Krishnamurthi, 1959) and in other countries have shown the relationship of tobacco with oral and oropharyngeal cancers. In the present study site analysis of those cases who did not take tobacco (Fig. 8 and 9) showed that the anterior $\frac{2}{3}$ of the tongue was the commonest site of the lesion, and buccal mucosa was an infrequent site. In contrast, the tobacco users (chewers, smokers or both) showed a high proportion with cancer of the buccal mucosa. In the former cases, the tumour was more frequently found on the lateral borders than the tip or the dorsum of the tongue. This may be due to exposure of the lateral border to constant irritation by the teeth especially when the latter were unhealthy. The mobile anterior part of the tongue was also more liable to suffer from trauma which may predispose it to the development of cancer more frequently than the dorsum or posterior portion.

The greater susceptibility of buccal mucosa to cancer in cases of tobacco users leads one to think of the possibility of this mucous membrane being more vulnerable to the possible carcinogenic effect of tobacco, pure or mixed with other ingredients. In chewers, it is understandable that this may be due to its maximum contact with raw tobacco and its other ingredients. The other possible explanation could be that when tobacco is chewed or smoked, its noxious agents (?carcinogenic) get dissolved in saliva. Normally some saliva remains constantly in the vestibule of the mouth, and may facilitate greater and prolonged contact of tobacco with the buccal mucosa. This high susceptibility of buccal mucosa to tobacco could be an important facet of study. However, the same predisposition of this site to cancer was also observed in smokers, though not to the same degree as the chewers. Is it that the buccal mucous membrane is more vulnerable to the action of tobacco whatever may be the mode of use?

Study of the site distribution of cases of oral leukoplakia (Fig. 10) showed that the high incidence of this lesion in buccal mucosa was also associated with the tobacco habit (86·3 per cent).

Association of smoking habit and cancer of the posterior part of the tongue, palate and tonsils has been observed by many workers (Niblock, 1902; Khanolkar, 1950). In the present study the number of cases of cancer of posterior $\frac{1}{3}$ tongue, palate and tonsils in either sex were not adequate to be subjected to statistical analysis. In males taking all habits together these were significantly related to cancers at all sites with a $\chi^2$ value of 511·8 ($P < 0{\cdot}05$). Both chewing and smoking were found to be significantly associated with cancers of buccal mucosa and anterior $\frac{2}{3}$ tongue. In females taking all habits together, there was a significant association of these to cancers of all sites with a $\chi^2$ value of 253·6 ($P < 0{\cdot}05$).

Reddy and Kameshwara Rao (1957) showed that in Andhra Pradesh the reverse smoking of cigars (with lighted end inside the mouth) is responsible for a high incidence of palatal cancer because both tobacco tar and heat work together in carcinogenesis. This was further supported by experimental work (Reddy *et al.*, 1960). It is possible that the sites of oral and oropharyngeal cancers in different types of smoking habit, such as cigar, pipe or bidi largely depend on the location of mucous membrane to get the first impact of the smoke which carries the greater part of both the burnt products of tobacco and heat.

In Europe and America cancer of the lip ranks first and it is thought to be particularly related to cigar and pipe smoking (Ebenius, 1943 ; Levin *et al.*, 1950 ; Moore *et al.*, 1953 ; Sadowsky *et al.*, 1953 ; Wynder *et al.*, 1957a). Cigar and pipe are rarely used by common people of India. Bidi (local cigarette) is commonly used in India by low income group people.

A close association of oral and oropharyngeal cancer and poor economic status of the individual has been noted amongst the cases. A majority of them could not afford balanced meals. Intake of spices had no relation to the occurrence of oral and oropharyngeal cancer. It is thought that both poor nutrition and the retention of tobacco quid in the oral cavity for a longer period of time enhances the carcinogenic process.

The study of serum vitamin A showed that 76·2 per cent of cases had subnormal levels. This low serum vitamin A is supposed to be an effect of improper nutrition. However, one has to keep in mind the possibility of hypovitaminosis A due to the presence of tumour itself. Vitamin A deficiency causes an increased keratinisation of oral mucous membrane like any other part of the body (Wahi *et al.*, 1962). This hyperkeratotic state of mucous membrane might be more vulnerable to the action of irritants and help in carcinogenesis (Wahi *et al.*, 1962). In such cases the low incidence of clinical manifestation of vitamin A deficiency may be explained on the basis of the fact that symptoms usually follow a decrease of vitamin A to the level of starvation.

A high coincidence of syphilis and cancer of the tongue has been reported in the past (Lund, 1938). Recently the cases in which this association is found have been decreasing. Trieger *et al.* (1958) found evidence of syphilis in 18 per cent of his cases. Syphilitic infection in the present study was encountered only in 10·9 per cent of cases. It was found to be least associated with carcinoma of buccal mucosa.

Alcohol has been incriminated as one of the major causes of oral cancer by Western workers (Wynder *et al.*, 1957b ; Trieger *et al.*, 1958). In the present study, alcohol had little association with these tumours.

The majority of the patients (87·6 per cent) in the present study had poor oral hygiene. It might be due to the effect of oral cancer, or the tobacco habit could be primarily responsible for it. Poor oral hygiene induces inflammatory changes in the oral mucous membrane which is supposed to be one of the factors responsible for oral cancer. Missing teeth resulting in faulty occlusion were noted in 49·7 per cent of cases. This might have been responsible for constant irritation of the oral mucosa. The presence of stumps and the use of artificial dentures also results in constant trauma of the mucous membrane. However, an associated tobacco habit makes it rather difficult to judge the individual role of oral hygiene and dental trauma in the causation of oral cancer.

### SUMMARY

One thousand nine hundred and sixteen cases of oral and oropharyngeal cancer have been studied. These tumours constitute 31·9 per cent of all malignant tumours. Buccal mucous membrane was the commonest site of involvement

(52·3 per cent). Males were more affected than females (2·3 : 1). Proportionately females suffered more from carcinoma of buccal mucous membrane and males from carcinoma of tongue, palate and tonsils. Sex predilection for different sites was related to the difference in tobacco chewing and smoking habits in male and female cases. The chewing habit was predominant in female patients and in males chewing and smoking were of equal importance. Tobacco was used in the majority of cases. Only 9·2 per cent of cancer cases were not addicted to tobacco use, while 66·5 per cent of controls did not use tobacco. The anterior $\frac{2}{3}$ of tongue was the commonest site of involvement in patients who did not use tobacco. Both in chewers and smokers buccal mucosa was the commonest site of lesion. Oral and oropharyngeal tumours were mostly seen in people of low socio-economic group. Low serum vitamin A was found in 76·2 per cent cases ; it was considered as an adjuvant in the carcinogenic process. Syphilis and alcohol showed no association with oral and oropharyngeal cancers. Poor oral hygiene was thought to be a contributory factor in the causation of oral cancer.

The present study reveals that environmental factors, specially the use of tobacco, play an important role in the aetiology of oral and oropharyngeal cancers in India.

The study was partly financed by a grant from the U.P. State Medical Research Council.

### REFERENCES

ACKERMAN, L. V. AND DEL REGATO, J. A.—(1962) 'Cancer Diagnosis, Treatment and Prognosis', 3rd Edition, St. Louis, U.S.A. (C. V. Mosby Co.).
BALA RAM, A. P.—(1902) *Indian med. Gaz.*, **37**, 414.
BROSS, I. J.—(1958) *Biometrics*, **14**, 18.
CADE, S.—(1950) *Am. J. Roentg.*, **63**, 716.
EBENIUS, B.—(1943) *Acta Radiol.*, Suppl., **68**, 1.
HALDAR, P. K.—(1953) *Indian J. Radiol.*, **7**, 13.
KHANOLKAR, V. R.—(1944) *Cancer Res.*, **4**, 313.—(1950) *Acta Un. int. Cancr.*, **6**, 881.
LEVIN, M. L., GOLDSTEIN, H. AND GERHARDT, P. P.—(1950) *J. Am. med. Ass.*, **143**, 336.
LUND, C. C.—(1938) *Surgery Gynec. Obstet.*, **66**, 810.
MOORE, G. E., BISSINGER, L. L. AND PROCHL, E. C.—(1953) *J. Am. Geriat. Soc.*. **1**, 497.
NIBLOCK, W. J.—(1902) *Indian med. Gaz.*, **37**, 161.
ORR, J. M.—(1933) *Lancet*, ii, 575.
REDDY, D. G. AND KAMESWARA RAO, V.—(1957) *Indian J. med. Sci.*, **11**, 791.
REDDY, D. G., REDDY, D. S. AND RAO, P. R.—(1960) *Cancer, N.Y.*, **13**, 263.
SADOWSKY, D. A., GILLIAM, A. G. AND CORNFIELD, J.—(1953) *J. natn. Cancer Inst.*. **13**, 1237.
SANGHVI, L. D., RAO, K. G. M. AND KHANOLKAR, V. R.—(1955) *Br. med. J.*, i, 1111.
SHANTA, V. AND KRISHNAMURTHI, S.—(1959) *Br. J. Cancer*, **13**, 381.
TRIEGER, N., SHIP, I. I., TAYLOR, G. W. AND WEISBERGER, D.—(1958) *Cancer, N.Y.*, **11**, 357.
WAHI, P. N., ARORA, S., SRIVASTAVA, M. C., KEHAR, U. AND BODKHE, R. R.—(1961) *Indian J. Path. Bact.*, **4**, 189.
WAHI, P. N., BODKHE, R. R., ARORA, S. AND SRIVASTAVA, M. C.—(1962) *Indian J Path. Bact.*, **5**, 10.
WAHI, P. N., LAHIRI, B., KEHAR, U. AND ARORA, S.—(1965) *Br. J. Cancer*, **19**, 628.
WAHI, P. N., SAXENA, S. N. AND WAHI, P. N.—(1958) *J. Indian med. Ass.*, **31**, 309.
WYNDER, E. L., BROSS, I. J. AND FELDMAN, R. M.—(1957b) *Cancer, N.Y.*, **10**, 1300.
WYNDER, E. L., HULTBERG, S., JACOBSON, F. AND BROSS, I. J.—(1957a) *Ibid.*, **10**, 470.